# The behavioural effects of the serotonin 1A receptor agonist buspirone on reward processing in healthy volunteers: A randomised, placebo-controlled study

**DOI:** 10.1177/02698811261430527

**Published:** 2026-03-28

**Authors:** Alexander L. W. Smith, Sorcha Hamilton, Susannah E. Murphy, Philip J. Cowen, Catherine J. Harmer

**Affiliations:** 1 Department of Psychiatry, Warneford Hospital, University of Oxford, UK; 2 Oxford Health NHS Foundation Trust, UK; 3 Department of Psychology, University of Bath, UK

**Keywords:** 5-HT1A, learning, motivation, reward, serotonin

## Abstract

**Background:**

The 5-HT_1A_ receptor is widely expressed throughout the brain and is implicated in reward processing; however, the acute effects of 5-HT_1A_ receptor agonism on reward and aversive processing in humans remain unclear.

**Aims:**

We investigate whether acute 5-HT_1A_ receptor agonism influences various stages of reward and aversive processing, specifically consummation, motivation and learning.

**Methods:**

In a double-blind, placebo-controlled study using a single 20 mg dose of buspirone, a serotonin 5-HT_1A_ receptor partial agonist, 62 healthy volunteers underwent 3 tasks: a taste task, an effort-expenditure decision task and a probabilistic instrumental learning task (PILT).

**Results:**

In healthy volunteers, acute buspirone increases the aversiveness of bitter tastes, non-significantly reduces willingness to exert effort and increases optimal choice during loss trials in the PILT.

**Conclusions:**

Speculatively, this could indicate that acute 5-HT_1A_ agonism may worsen several stages of aversive processing, with minimal effect on reward processing. The study was pre-registered (clinicaltrials.gov, Serotonin-receptor Agonism in Reward Processing, NCT05357547).

## Introduction

5-HT_1A_ receptors are implicated in the pathophysiology of depression through several lines of evidence, including post-mortem, pharmacological and neuroimaging studies (Drevets et al. 1999; Savitz et al., 2009). The 5-HT_1A_ receptor is widely distributed throughout the brain (Ito et al., 1999), and a reduction in binding in the raphe and cortical regions has been observed in depression (Drevets et al., 2007; Sargent et al., 2000). Furthermore, a healthy volunteer PET study demonstrated a positive correlation between 5-HT_1A_ binding in the hippocampus and reward sensitivity in a probabilistic decision-making task (Faulkner et al., 2014). Additionally, 5-HT_1A_ binding in the raphe nucleus has been shown to positively correlate with orbitofrontal cortex activation when viewing rewarding images (Hahn et al., 2009).

It is important to clarify the effects of 5-HT_1A_ receptors on reward processes because manipulation of this receptor may have utility in the pharmacotherapy of depression, including anhedonia. Evidence for this includes 5-HT_1A_ autoreceptor antagonism expediting the response to **Selective Serotonin Reuptake Inhibitors** (SSRIs) (Portella et al., 2011), and 5-HT_1A_ receptor agonism are involved in the clinical effects of the licensed multimodal antidepressant vortioxetine (D’Agostino et al., 2015), a medication that has shown efficacy in treating anhedonia (McIntyre et al., 2021). There is increasing consensus that 5-HT_1A_ receptors have a role to play in antidepressant treatments (Blier and Ward, 2003; Celada et al., 2013).

A number of functional measures have been used to indicate 5-HT_1A_ receptor engagement in pharmacological challenge studies, such as a hypothermic response and endocrine response, specifically increases in the hormone ACTH, with a subsequent increase in cortisol. The increase in cortisol following 5-HT_1A_ receptor agonism has been frequently observed (Cowen, 1993; Savitz et al., 2009; Pitchot et al., 2002) and thought to be attributable to post-synaptic 5-HT_1A_ agonism (Pan and Gilbert, 1992). The location of the 5-HT_1A_ receptors involved in the hypothermic response to buspirone in humans is more contested, but some involvement of 5-HT_1A_ auto-receptors is likely (Young et al., 1994).

Converging pre-clinical evidence indicates that 5-HT_1A_ receptor agonism can improve reward behaviours (Hayes and Greenshaw, 2011; Kinney et al., 1998). Furthermore, 5-HT_1A_ receptors appear to play a role in the antidepressant effect of the rapidly acting antidepressant ketamine in the forced swim test model of depression (Fukumoto et al., 2018). Specifically, blocking the 5-HT_1A_ receptor attenuates the antidepressant effect of ketamine while cortical injection of a 5-HT_1A_ agonist mimics the action of ketamine in this depression model (Fukumoto et al., 2018).

The aforementioned studies examining the role of 5-HT_1A_ receptors in reward are animal experimental studies. However, currently, there are no reported human studies directly probing the 5-HT_1A_ receptor in reward processing or anhedonia.

This study explored whether direct 5-HT_1A_ receptor agonism influenced consummation of primary taste stimuli; motivation through willingness to exert effort for reward; and reward and aversive learning, all key stages in reward processing (Berridge et al., 2009; Husain and Rosier, 2018). We used a single dose of the 5-HT_1A_ receptor partial agonist, buspirone 20 mg, in a between-subject, placebo-controlled study and measured performance on three different tasks designed to probe differing stages of reward processing. These included subjective ratings of the pleasurableness of four different tastes, an effort-discounting decision-making task, and a probabilistic instrumental learning task (PILT).

We hypothesized that acute 5-HT_1A_ receptor agonism would improve subjective pleasurableness of rewarding tastes, increase willingness to exert effort for reward, and increase reward learning. Additionally, we predicted that 5-HT_1A_ receptor agonism would not have a strong effect on the processing of aversive stimuli, for example, experiencing bitter tastes, willingness to exert increased effort or learning and sensitivity to loss outcomes.

## Methods

### Subjects and study design

The study was part of a larger study, of which details regarding participant selection and design have been described elsewhere (Smith et al., 2025). Briefly, a double-blind, placebo-controlled study recruited 62 healthy volunteers (31 participants received a single dose of buspirone 20 mg and 31 participants received a placebo, both identically encapsulated, stored in individual envelopes labelled with sequential intervention ID). A 20-mg dose of buspirone was selected as it is within the nationally recommended treatment range (Joint Formulary Committee, 2025). This dose provides activation of both pre- and post-synaptic 5-HT_1A_ receptors in human experimental models (Cowen et al., 1994) with good tolerability for participants (Bernasconi et al., 2015; McAllister-Williams and Massey, 2003).

Sample size calculation, inclusion exclusion criteria and **Consolidated Standards of Reporting Trials** (CONSORT) checklist is detailed in the Supplemental material (Supplemental text 1; Supplemental Table 1). Participants aged between 18 and 65 years were block randomised (by a staff member not involved with the study using an online randomisation tool) into two groups, stratified by gender. Study personnel completed screening, enrolment and administration of interventions according to a blinded study allocation list. Randomisation of study medication was recorded on a master list, withheld from all members of the study team until unblinding. Allocation of the study medication was recorded on a Randomisation List, which was updated when each new participant entered the study.

The study was approved by the Local Research Ethics Committee (Oxford, MSD-IDREC reference R79236/RE006). Written informed consent was obtained from all participants.

Participants were recruited through community and social media advertising and underwent a medical and psychiatric screening before enrolment to ensure they were suitable for the study in relation to inclusion and exclusion criteria as described elsewhere (Smith et al., 2025). All data were collected from May 2022 until the final participant completed the study in April 2023 at the Department of Psychiatry, Warneford hospital, University of Oxford.

### Study tasks and outcome measures

Participants underwent three tasks (timing post-intervention in parentheses): an effort-grip task (60 minutes), a taste task (100 minutes) and a PILT (150 minutes).

The taste task required subjects to rate the intensity, anticipation of pleasure and actual pleasure of four separate tastes (sweet, sour, salty and bitter) on a visual analogue scale (0–100). The tastes were administered from a validated commercial test kit (Taste Strips, Burghart Messtechnik, Holm, Germany) and chosen for suprathreshold concentrations of taste to increase the likelihood of detection. Participants were informed what taste they were being given. To mitigate individual differences in taste preference, participants underwent the task twice: pre-intervention and post-intervention. The outcome measure for the task was the actual pleasure rating of each task. Taste tasks have been widely used in testing the effect of pharmacological interventions in healthy volunteers (Kaltenboeck et al., 2022; McCabe et al., 2010; Smith et al., 2021). Whilst these studies may not have consistently identified differences between groups in terms of pleasantness, anhedonia-specific scores were found to negatively correlate with pleasantness and detection threshold in a depressed patient sample, indicating its potential utility in assessing consummatory reward processing (Berlin et al. 1998). The effort grip task is used here as described by (Attaallah et al., 2024) and performed once post-intervention. After a short practice trial subjects underwent a decision phase whereby an offer was presented comprising of a hypothetical number of apples (1, 4, 7, 10 or 13 – five levels of reward) in exchange for physical effort (16%, 32%, 48%, 64% and 80% of individual maximal voluntary contraction – five levels of effort), resulting in 25 unique offers. Participants could either accept or decline the offer by selecting Yes or No. This was repeated a further 4 times giving 125 trials in total (5 trials of each 25 unique offers). In the work phase of the task, participants were informed that 10 trials from the previous 125 trials would be chosen at random for them to attempt (i.e. exert physical effort sufficient to ‘win’ the apples in the offer). Only if the participant accepted an offer in pre-determine trials (These were 5% × 16%; 3% × 48%; 2% × 64%; 4% × 32%; 4% × 16%; 2% × 16%; 1% × 16%; 3% × 32%; 2% × 64% and 4% × 80%), would they be presented with the opportunity to exert effort for the reward. Participants were informed that they could earn extra money depending on the number of apples they collected in the work phase, but all were given the same final amount of money. Stimulus presentation was programmed in MATLAB (The MathWorks, Inc., USA). Force was recorded using a BioPac M46 and SS25LB hand dynamometer (BIOPAC Systems, Inc., USA). The primary outcome measure was the proportion of offers accepted. A secondary outcome measure was the reaction time from offer presentation to effort expenditure. Effort-tasks of this nature have previously demonstrated that increasing effort-sensitivity positively correlates with apathy, with associated neural correlates in healthy volunteers (Bonnelle et al., 2016). Furthermore, it also distinguishes between individuals experiencing depression and healthy controls (Cléry-Melin et al., 2011). Finally, effort grips tasks are sensitive to serotonergic manipulation in healthy volunteers (Meyniel et al., 2016) and are used in commercial drug development (Bilderbeck et al., 2020).

The PILT, originally described by Pessiglione et al. (2006), was performed once, post-intervention. The task required participants to choose one symbol in a pair of symbols with the aim of either winning or avoiding losing money (20 p). One pair is associated with a win of 20 p or no change, the other pair loss of 20 p or no change. Each symbol pair is associated with reciprocal probabilities of that outcome (0.7 or 0.3). Each run consisted of 30 interleaved win and loss trials, and participants underwent three runs (each run starting with new symbols to learn). The outcome measure was the proportion of participants choosing the correct symbol in win and loss trials. Behaviour in this task captures reinforcement well, reflecting updating expected values and prediction error (Pessiglione et al., 2006), which is dysfunctional in depression (Halahakoon et al., 2020; Walsh et al., 2018) as well as sensitive to monoaminergic manipulations in humans (Halahakoon et al., 2024; Murphy et al., 2020; Pessiglione et al., 2006; Walsh et al., 2018).

This study formed part of a larger investigation in which temperature and salivary cortisol were recorded at 30-minute intervals. Details regarding collection and analysis have been published previously (Smith et al., 2025).

### Statistical analysis

To assess whether buspirone influenced pleasure ratings, independently of baseline preferences, a series of linear regression model were built for each taste. The primary outcome was post-drug pleasure ratings and candidate predictor variables included pre-drug pleasure rating, post-drug intensity and anticipation ratings, post intervention nausea and allocation. For each taste, several linear regression models were compared to find the most parsimonious model with the best fit. This was based on Akaike Information Criterion (AIC), Bayesian Information Criterion (BIC) and *R*^2^ values. Model comparison to determine the most appropriate models is reported in Supplemental material (Supplemental Table 3).

The effort-based task used the overall proportion of offers accepted between groups, which was compared using an unpaired *t*-test. The proportion of offers accepted within each effort level or reward level was calculated for each participant, and separate mixed effects linear regressions were performed (i.e. one for effort, one for reward and one for effort and reward). To explore the relationship between raw offer acceptance and group at each unique effort × reward combination (e.g. a binary outcome of accept or decline), a logistic regression was also performed for the whole dataset with fixed effects of reward, effort and allocation group and reward and effort as within-subject random factors. All interactions between reward, effort and allocation were included. To visualise the differences in acceptance of an offer, using the full mixed effects logistic regression (which included random effects of subject, in order to account for individual differences – see Supplemental text 2 for model rationale), log odds of accepting an offer were calculated at the trial level for each participant. Any significant effects were followed up with pairwise comparisons between allocation groups using estimated marginal means (corrected).

Reaction time for offer decision was explored using mean log-transformed reaction times in a repeated measures ANOVA was performed with allocation, reward and effort level as factors.

For the reward learning task, an average optimal choice for each trial for each participant was calculated (participants underwent three runs of the task; this value was between 0/3 and 3/3). The average optimal choice for each trial for each participant was used to calculate the group mean at each trial. Data were separated into win and loss conditions, then the mean optimal choice in each trial (1–30) was compared between groups using a two-way ANOVA with allocation, trial number, and their interaction as fixed effects, which were reported as *F* statistics and *p*-values. After visual inspection of learning graphs, a trial cut-off was established after which the optimal choice had plateaued. The above analysis was repeated using only trials after this cut-off as a proxy measure to examine the effect after learning.

ANOVA results are reported as *F* statistics and *p*-values, and regression results are reported as beta coefficients and *p*-values.

Side effect data were collected for participants as part of a larger study, of which this work was a part of, and is reported in previously published (Smith et al., 2025).

#### Data cleaning

Where participants could not register a taste (intensity = 0), this was removed from the specific taste dataset. This occurred for three participants, all of whom were allocated to the buspirone group. Owing to data collection error, nausea data for two participants in the buspirone group were not collected. When nausea was included as a covariate, data from these two participants were removed.

Similar to previous studies using the effort-based task (Saleh et al., 2021), trials with reaction time < 0.4 seconds were deemed accidental, and for each participant, trials with reaction times above three standard deviations above the participant’s mean reaction time were removed. This equated to 2.4% of all trials.

**Figure 1.**
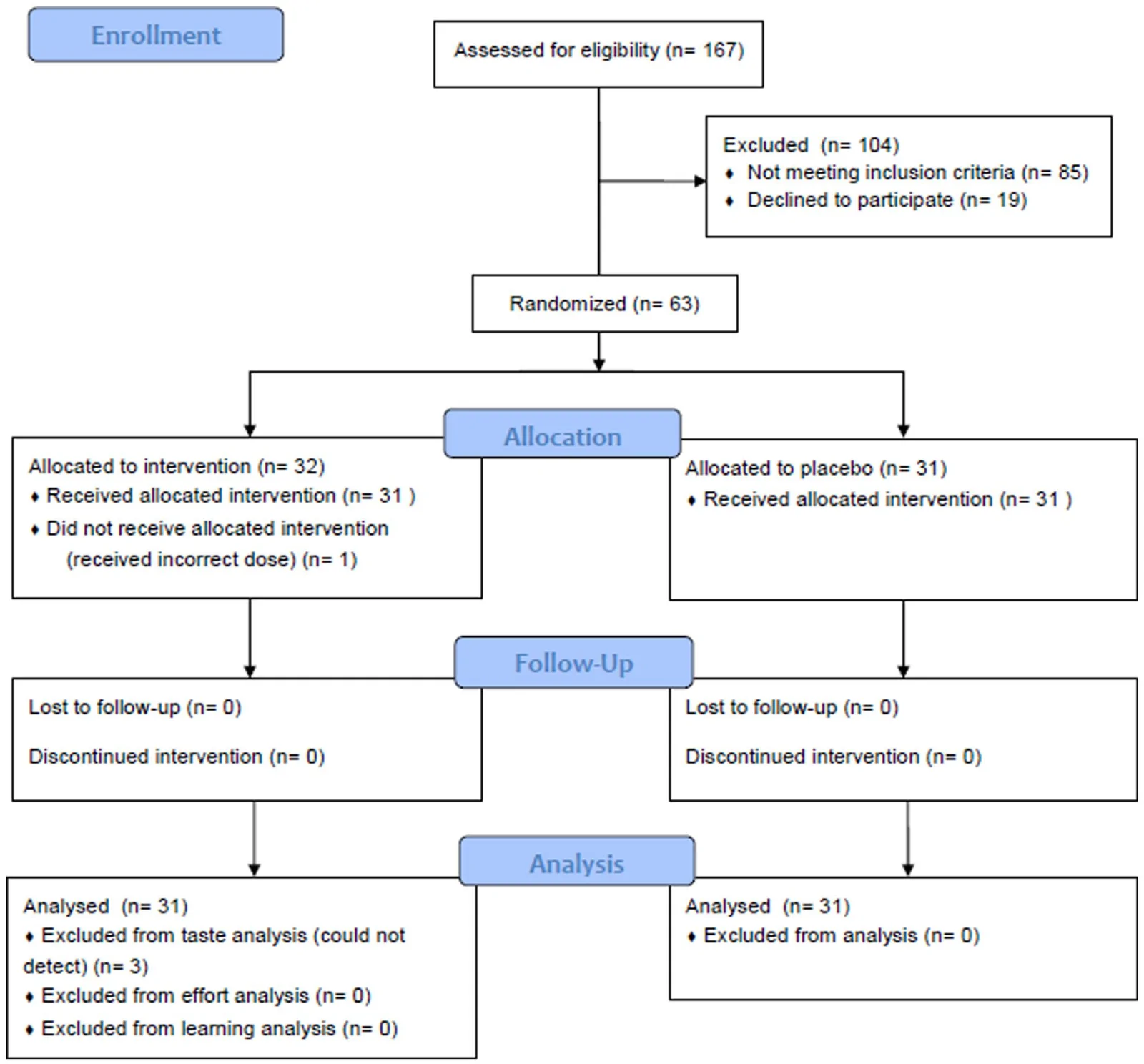
CONSORT flow diagram.

In the reward learning task, 12 trials (0.1%) were particularly fast (<0.2 second) and were removed on the presumption of it likely being an error. See Figure 1 for the CONSORT flow chart.

## Results

Groups were well matched for age, gender and in baseline Beck’s Depression inventory scores (Supplemental Table 2).

As reported previously, acute buspirone administration, relative to placebo, resulted in significant increases in salivary cortisol from 90 minutes post challenge onwards (Smith et al., 2025). However, no such difference was observed in temperature response. These findings confirm engagement of post-synaptic 5-HT_1A_ receptors by buspirone during the study.

### Taste task

Separate linear regression analyses for each taste type revealed no significant main or interacting effect of allocation on post-intervention pleasurableness of sweet, sour or salt tastes (*p* > 0.1). In analysis of the post-intervention pleasurableness of bitter tastes a main effect of allocation (β = 7.56, *p* = 0.039; η_p_^2^ = 0.09, 95% CI = 0.00–1.00) was observed, indicating that the buspirone group found bitter tastes more aversive than the placebo group, that is, the buspirone group found the bitter taste worse by 7 points (for descriptive statistics see Supplemental Table 4).

### Effort expenditure task

**Figure 2.**
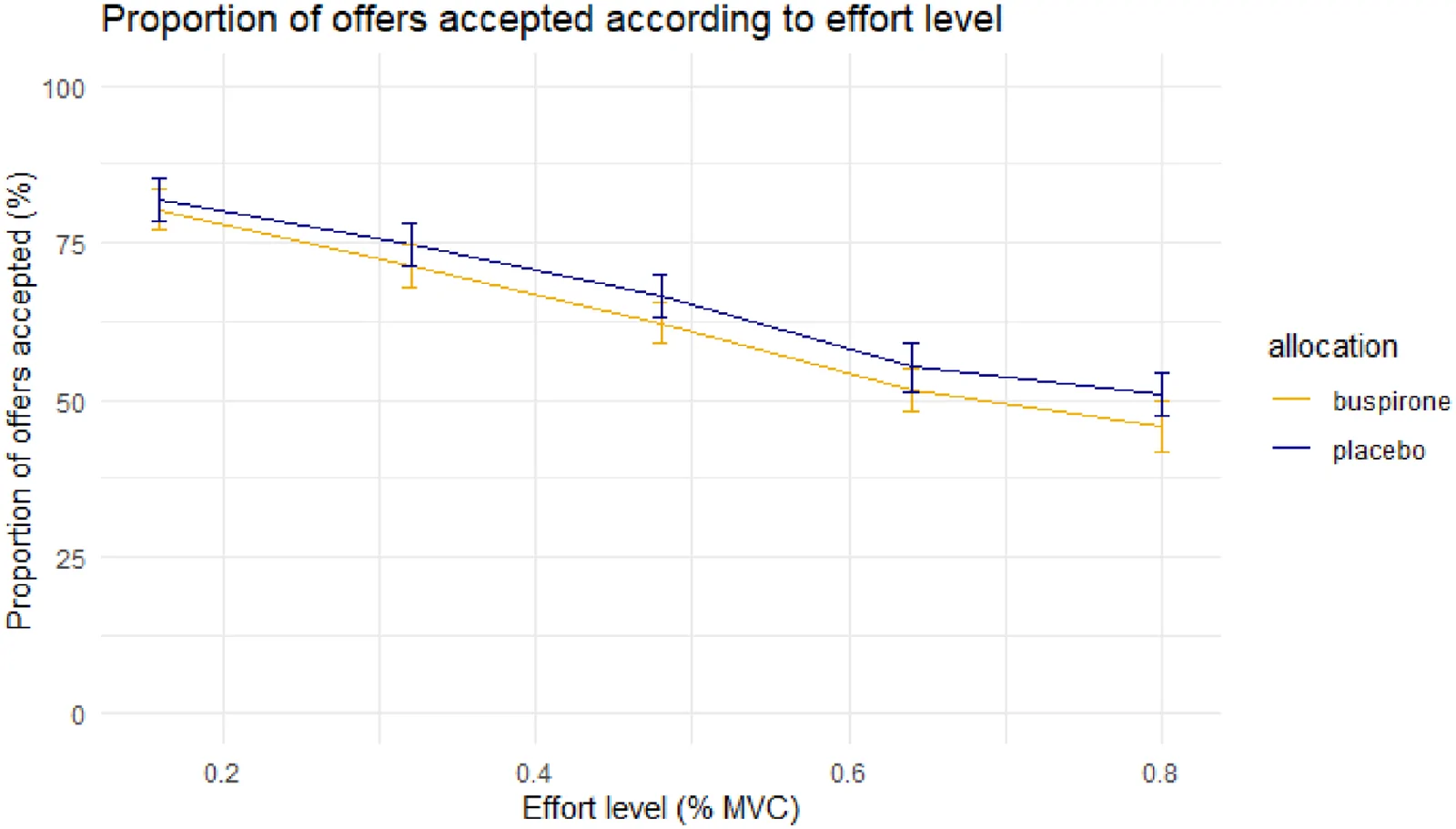
Mean proportion of offers accepted at each effort level between groups. Error bar = ±1 S.E.M.

**Figure 3.**
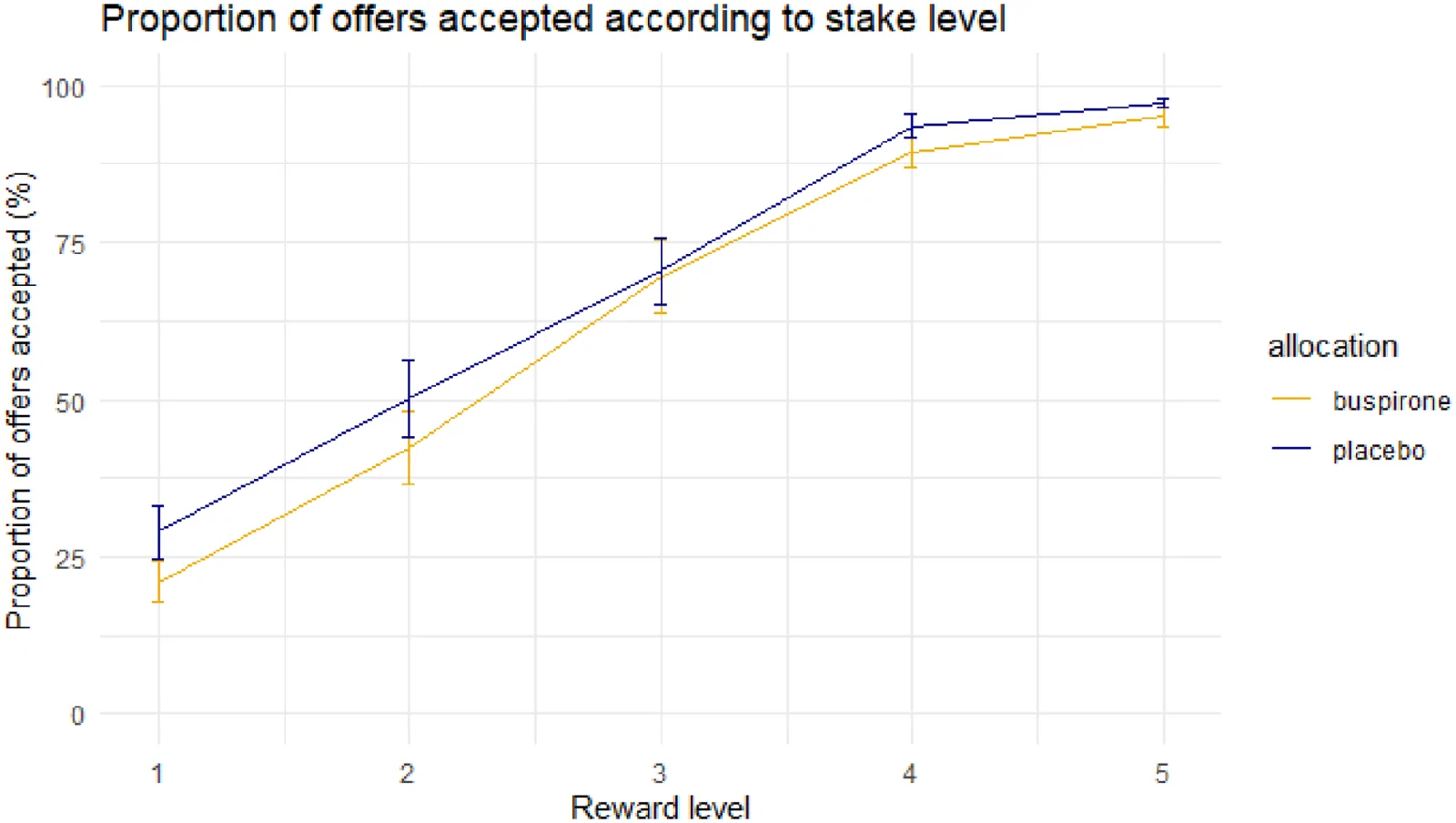
Mean proportion of offers accepted at each reward level between groups. Error bar = ± 1 S.E.M.

No significant difference between groups in overall acceptance rate was identified (*t*(47) = −0.83, *p* = 0.41; buspirone = 62%, placebo = 66%). Mixed effects linear regression analyses demonstrated no significant main or interaction effect of allocation (*p* > 0.1; Figures 2 and 3).

#### Comparison at the level of effort and reward

The interaction effect of effort and reward was explored by dividing each offer into its unique reward × effort combination (25 levels in total derived from 5 levels of reward and 5 levels of effort). Mixed effects linear regression of the proportion of offers accepted for each unique effort * reward offer indicated no significant main or interaction effect of allocation (*p* > 0.1).

**Figure 4.**
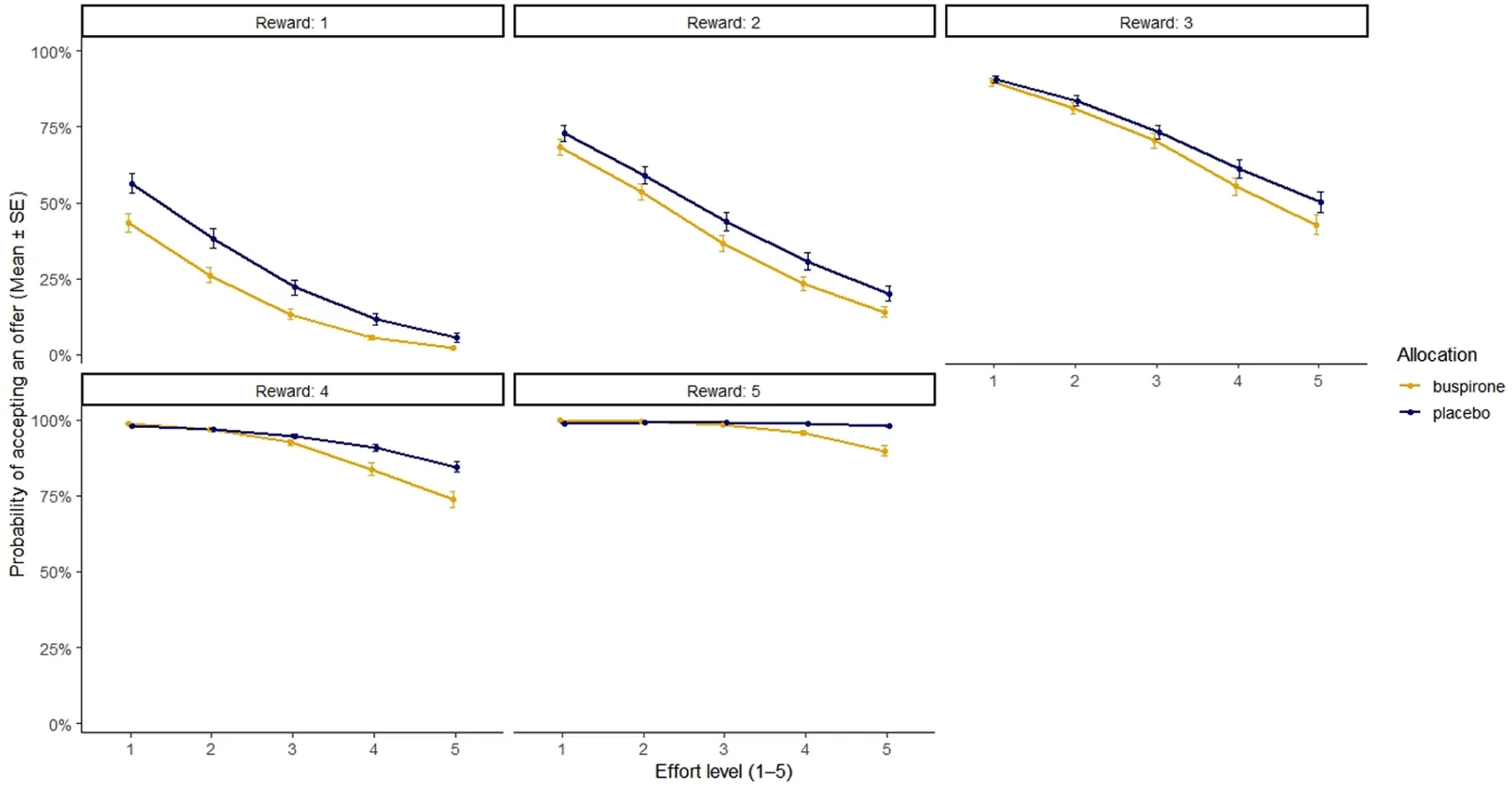
Scatter plot of the mean probability of accepting an offer at each reward and effort level between groups. Mean probability calculated using estimated marginal means from the full mixed effects logistic regression model. Error bars = ±1 S.E.M.

The mixed-effects logistic regression, examining the binary outcome of accepting an offer or not, indicated that the main effect of allocation was not significant (β = 0.35, *p* = 0.50); however, a significant three-way interaction between reward level, effort level, and allocation on the likelihood of accepting an offer was observed (β = 0.56, *p* = 0.031). The odds ratio of accepting an offer in the placebo group, compared to the buspirone group, was 1.74, 95% CI: 1.05–2.89. To visualise the significant three-way interaction between reward level × effort level × allocation, the probability of accepting each unique effort × reward offer for each allocation group (derived from the mean ± 1 S.E.M. of log odds of accepting an offer from the mixed effect logistic regression model) was calculated (Figure 4).

Despite the significant reward * effort * allocation interaction, pairwise contrasts using estimated marginal means, and corrected for multiple comparisons (Holm), revealed no significant differences emerged between allocation groups at any of the 25 unique effort * reward combinations.

No significant main or interaction effect of allocation on log-transformed reaction times was observed (*p* > 0.1).

### Reward learning task

**Figure 5.**
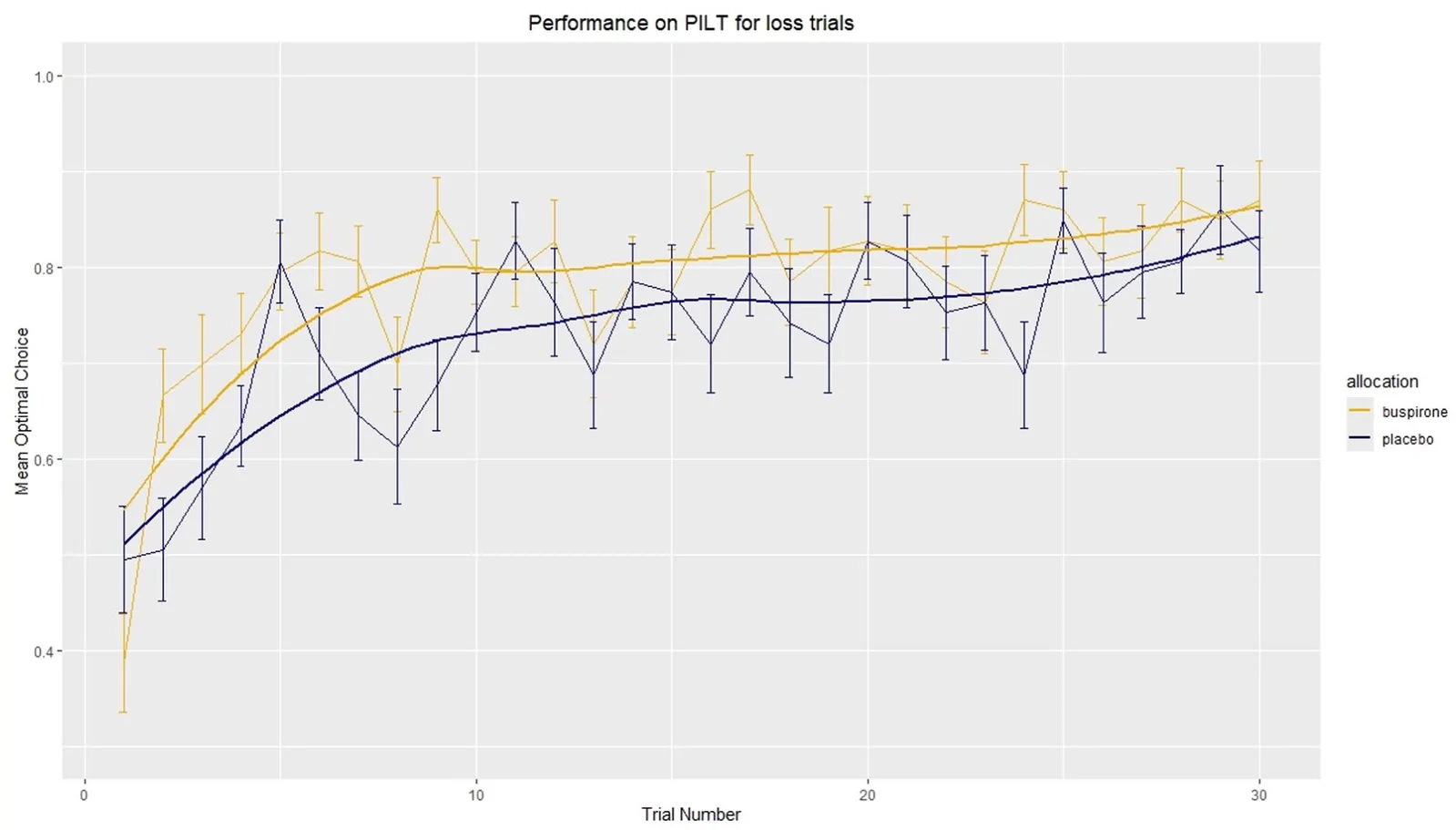
Mean optimal choice in loss trials. Mean optimal choice was calculated by subjects choosing the symbol least likely to lose in a loss trial. The number of optimal choice trials was divided by total loss trials for each participant to give the proportion of optimal choices at each trial (e.g. two runs, therefore optimal choice can be made on both, one or neither trial, giving a proportion of either 0, 0.5 or 1). The mean of proportion of optimal choices was then calculated for each group. Error bars = ±1 S.E.M. \n Line of best fit uses Locally Estimated Scatterplot Smoothing (LOESS) function in geom_smooth() in R.

**Figure 6.**
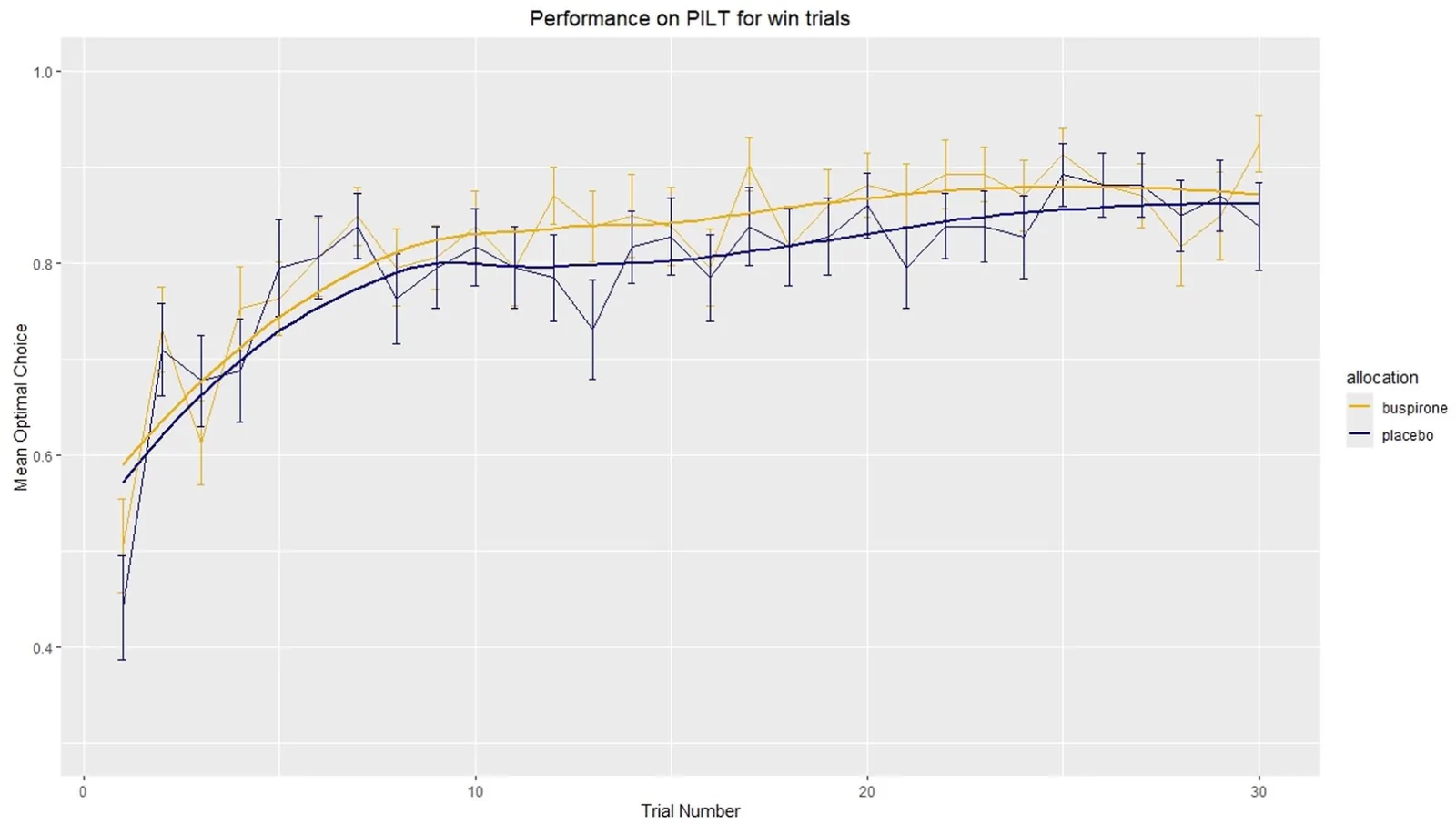
Mean optimal choice in win trials. Mean optimal choice was calculated by subjects choosing the symbol most likely to win in a win trial. The number of optimal choice trials was divided by total win trials for each participant to give the proportion of optimal choices at each trial (e.g. two runs, therefore optimal choice can be made on both, one or neither trial giving a proportion of either 0, 0.5 or 1). The mean proportion of optimal choices was then calculated for each group. Error bars = ±1 S.E.M. \n Line of best fit uses Locally Estimated Scatterplot Smoothing (LOESS) function in geom_smooth() in R.

In the loss condition, a significant difference in optimal choice was found between allocation groups (*F*_(1, 56)_ = 9.08, *p* = 0.0039, η_p_^2^ = 0.14, 95% CI: 0.03–1.00; Figure. 5). In corresponding analysis, no significant difference was observed in the win condition (*F*_(1, 56)_ = 2.49, *p* = 0.12; Figure 6). Visualisation of optimal choice indicates that for both win and loss conditions, the buspirone group shows an increased number of optimal choices compared to the placebo group, especially when it appears learning has plateaued after trial 10.

Analysis of mean optimal choice after trial 10 was performed and indicated a significant difference between groups exist in both the win (*F*_(1, 38)_ = 11.23, *p* = 0.0018, η_p_^2^ = 0.23, 95% CI: 0.06–1.00) and loss (*F*_(1, 38)_ = 10.28, *p* = 0.0027, η_p_^2^ = 0.21, 95% CI: 0.05–1.00) conditions.

No significant main effect of allocation or interaction effect of allocation and condition was observed for log-transformed reaction times (*p* > 0.1).

## Discussion

Acute buspirone appears to increase the aversiveness of bitter tastes, affecting both the intensity and anticipation of the taste experience. Furthermore, acute buspirone maintains effort discounting in an effort-expenditure task at high reward levels. Finally, buspirone appears to improve optimal choice selection in a PILT involving loss and reward. To the best of our knowledge, these are novel findings examining the effect of 5-HT_1A_ receptor agonism on these models of reward processing in humans.

The principal actions of acute buspirone administration are full agonism at 5-HT_1A_ autoreceptors and partial agonism at post-synaptic 5-HT_1A_ receptors (Loane and Politis, 2012). This would be expected to diminish serotonin release from presynaptic serotonergic neurones and thus decrease overall serotonin transmission, particularly at synapses whose post-synaptic receptors are not of the 5-HT_1A_ subtype. In this context, as well as comparing with studies involving direct 5-HT_1A_ agonism, one could compare results presented here with those of acute tryptophan depletion (ATD), which would be expected to lower overall serotonin neurotransmission by decreasing serotonin release from presynaptic terminals, albeit to a modest and transient degree.

A study of 25 healthy female volunteers found that ATD led to the rating of bitter tastes as less pleasurable, compared to the placebo group, consistent with results presented here (Smith et al., 2021). Another investigation of 36 lean, healthy volunteers (BMI between 18.5 and 24.9) found that ATD, compared to placebo, led to a non-significant decrease in calories consumed through sweet foods and a reduction in first-choice sweet foods (Pagoto et al., 2009). This was interpreted as a decreased preference or salience of pleasurable sweet foods, which is broadly in agreement with results here (i.e. buspirone reducing the pleasurableness of tastes). In summary, this study adds to the evidence that indicates that in humans, reducing serotonergic transmission, through ATD or buspirone, increases the aversiveness of bitter tastes.

Results presented here indicate that acute 5-HT_1A_ agonism modifies subjects’ aversion to effort, thus the motivation in relation to the incentive offered. This supports some findings amongst a field of contrasting evidence. In support of our results, several within-subject studies, utilising a novel cued-reinforcement reaction time task, found that reaction times became faster as the probability of reward increased, an effect that was abolished by ATD, suggesting lowered serotonergic transmission reduces motivational vigour for high probability rewards (Cools et al. 2005; Roiser et al. 2006).

This finding is broadly consistent with results presented here, namely that reducing serotonergic transmission via buspirone administration maintains a sensitivity to effort that would otherwise diminish with increasingly rewarding outcomes in decision-making. However, it is noteworthy that no reaction time difference was found, indicating that the effects of buspirone on decision making may not convert into changes in motivational vigour. This could be owing to the studies mentioned used a within-subjects approach, which could have greater sensitivity to reaction time difference, as well as task difference, namely the aforementioned task had to discern between three shapes, was response time-dependent and with the outcome of winning a reward. In contrast, the task used here to measure motivation evaluated only one option at a time, deciding if an offer ‘is worth it’ to the individual, and the outcome was not dependent on reaction time. Therefore, reaction times here were possibly too long to detect group differences.

However, preclinical evidence indicates 5-HT_1A_ receptor agonism may enhance the motivation to exert effort. Using a response duration differentiation schedule, whereby rats had to learn to press a level for 1–1.3 seconds to receive a food reward, 8-OH-DPAT and buspirone 10 mg/kg i.p. increased the mean duration of responses (Kinney et al., 1998). The authors hypothesized that the effects of buspirone and 8-OH-DPAT in increasing the duration of responding could have been due to post-synaptic 5-HT_1A_ receptor agonism. 8-OH-DPAT is an effective post-synaptic 5-HT_1A_ receptor agonist, and the dose of buspirone was high. This may account for the discrepancy with results presented here, where the effects of acute buspirone may be primarily due to 5-HT_1A_ autoreceptor stimulation.

Furthermore, the effect of buspirone to reduce willingness to exert effort reported here is small and is not apparent from initial, grouped analysis. Therefore, this finding should be treated with caution. Nevertheless, in the context of increasing effort being aversive, the effort expenditure task results here are consistent with enhanced sensitivity to aversive tastes in the primary reward task.

In summary, results of acute buspirone maintaining the aversive effect of effort are indirectly supported by some ATD studies in humans, and contribute novel evidence to the field of serotonin and motivation. Thus reduction in serotonergic transmission impacts explicit effort-discounting decision making, an effect presumably mediated by diminished serotonin function impacting on activity in brain regions such as the striatum, frontal cortex and cingulate cortex (Delcourte et al., 2021).

Acute buspirone administration improved learning about aversive loss outcomes but not rewarding win outcomes in a PILT. These results are in contrast to a smaller, healthy volunteer study examining the influence of ATD on a two-arm bandit, gambling task, varying the magnitude and probability of reward. This previous study found ATD did not influence probabilistic choice of healthy volunteers between a high-probability small reward and a low-probability high reward (Anderson et al., 2003). However, this study examined reward only, varied reward magnitude and involved an element of gambling, which may confound results.

Whilst data involving instrumental probabilistic learning is sparse, much evidence exists examining the role of serotonin depletion and reversal learning in humans. One group used an observational reversal learning task and found that healthy volunteers made fewer prediction errors for punishment following ATD (Cools et al., 2008). Specifically, under baseline conditions, an increase in prediction errors for punishment trials, but not for reward trials, was observed. However, following ATD, this difference was abolished, with prediction error for both reward and punishment trials being similar. It was suggested that serotonergic transmission provides resilience to aversive outcomes or protective bias against anticipation of punishment; however, when serotonin activity is lowered, this bias is lost, and subjects have an enhanced ability to anticipate punishment (Cools et al., 2008). This is indirectly supported by recent findings in our group whereby acute buspirone appeared to have an anxiogenic and depressant effect, speculatively increasing sensitivity and anticipation for aversive outcomes (Smith et al., 2025).

These findings were replicated in a larger, between-subjects healthy volunteer study, which found that the increased sensitivity to prediction of punishment following ATD was not explained by changes in mood (Robinson et al., 2012).

In contrast to the findings presented here, a previous study found that ATD in healthy volunteers impaired reward learning, specifically by reducing sensitivity to reward. The study used a probabilistic 4-arm bandit task and found increased perseveration (or reduced flexibility) and reduced sensitivity to rewarding, but not aversive, stimuli in the ATD group (Seymour et al., 2012). These divergent findings could be due to differences in task effects. In Seymour et al. (2012), a four-arm bandit was used with reward and punishment (a painful stimulus) being delivered together, with varying probability. This makes it challenging to separate the effects of either outcome on learning, in that reward is perhaps more salient as it is compared to punishment rather than reward omission. Reward omission is more likely to reflect reality, for example, if you do not win the lottery, having bought a ticket, you do not fear receiving painful stimulus instead.

These findings can be considered alongside our recent findings examining the effect of acute buspirone dose on emotional processing and cognition in healthy volunteers (Smith et al., 2025). In that study, buspirone reduced accuracy for recognising facial expressions of disgust and increased misclassification of negative facial emotions as other negative expressions, interpreted as inducing a subtle negative bias in emotional processing (Smith et al., 2025). The present results indicate that acute buspirone treatment can increase the impact of aversive information across multiple domains: primary sensory experience (greater aversion to bitter tastes), effort-based decision making (maintained aversion to effort even at higher reward levels), and probabilistic learning (enhanced learning about loss outcomes relative to reward).

A tentative hypothesis could be that acute buspirone shifts information processing towards negative outcomes, while at the same time reducing the precision with which specific negative, emotional cues are discriminated. This manifests as reduced accuracy for disgust and increased ‘negative-to-negative’ misclassifications. However, here acute buspirone could lead to a stronger weighting of aversive cues (e.g. bitterness, effort, loss) in guiding subjective evaluation and learning. In summary, the results of the two studies could possibly indicate that a transient reduction in serotonergic transmission via 5-HT_1A_ auto-receptor activation may enhance the salience of negative or aversive signals across social, sensory and decision-making contexts.

It is widely accepted that serotonergic involvement in depression is unlikely to represent a simple chemical imbalance. However, serotonin neurons are known to exert widespread neuromodulatory influence on neural systems involved in psychological processes such as mood, emotion, motivation, reward and cognition, which are intimately associated the clinical syndrome of depression (Jauhar et al., 2023). Studies using the technique of tryptophan depletion have shown that low levels of brain serotonin by themselves do lead to depressive symptoms. However, when low brain serotonin occurs in the presence of other depressive vulnerability factors, clinical symptomatology can be induced (Smith et al., 1997). This suggests that serotonergic dysfunction may interact with pre-existing neurobiological risk factors and neural networks to produce depressive symptoms (Jauhar et al., 2023). In parallel, receptor-focused work highlights that serotonergic transmission is multifaceted, with multiple receptor subtypes producing distinct – and sometimes opposing – effects across brain regions (Köhler et al., 2016; Sharp and Barnes, 2020). Additional complexity can be introduced whereby the same receptor subtype may activate different intracellular pathway dependent on its location within the brain as well, a concept called ‘biased agonism’ (Newman-Tancredi et al., 2022). Together, this emphasises the importance of interpreting behavioural findings in terms of receptor- and circuit-level mechanisms rather than overall ‘more versus less serotonin’.

Within this lens, broader systems-level and computational accounts suggest that serotonin contributes to processes directly relevant to depression, including reward and punishment processing such as valuation and anticipation (Dayan and Huys, 2009; Höflich et al., 2019; Miyazaki et al., 2020). Current views propose that serotonergic firing broadly contributes to how the value of events is estimated, and such value-related computations are disrupted in depressed patients (Huys and Browning, 2022).

Our pattern of results following acute buspirone is somewhat consistent with this account. Acute buspirone is expected to act strongly at 5-HT_1A_ auto-receptors, reducing serotonin release and disrupting canonical serotonin signalling generally. In line with the above account (Huys and Browning, 2022), this may bias valuation and learning toward aversive outcomes, reflected here in increased bitterness aversiveness and enhanced learning/optimal choice on loss trials. It is important to stress, however, that this acute profile should not be assumed to mirror clinical effects of longer-term serotonergic treatments: receptor-level adaptations (including potential auto-receptor desensitisation with repeated dosing) and downstream plasticity may lead to different behavioural consequences over time with 5-HT_1A_ receptor agonists. Indeed, there is evidence that repeated treatment with buspirone has some efficacy in the treatment of major depression (Rickels et al., 1991).

### Limitations

An assumption underlying the interpretation of results here is that acute buspirone principally activates somatodendritic 5-HT_1A_ receptors, leading to a reduction 5-HT release in most terminal fields. However, we do not have direct evidence for this. Indeed, we found that this dose of buspirone in healthy participants increased saliva cortisol levels (Smith et al., 2025), suggesting some activation of post-synaptic 5-HT_1A_ receptors in the hypothalamus (Cowen et al., 1994; Pan and Gilbert, 1992). It is possible that a longer course of buspirone may lead to down regulation of somatodendritic 5-HT_1A_ receptors specifically (Sim-Selley et al., 2000), with the post-synaptic 5-HT_1A_ agonist action of buspirone thereby predominating. Thus, repeated administration of buspirone would enable a hypothetical comparison of the influence of pre- versus post-synaptic 5-HT_1A_ agonism on reward processing using an identical compound.

On a similar theme, behavioural tasks were administered sequentially over a period of 2.5 hours post-dose. Buspirone possesses a T_max_ of 40–90 minutes and *t*_1/2_ of 2–3 hours (Strides Pharma UK, 2022). It is therefore plausible that plasma buspirone concentration varied during the testing time; therefore, it cannot exclude the possibility that drug effects differed somewhat between earlier and later tasks. However, this is mitigated by all tasks being conducted within buspirone’s pharmacologically active window with a consistent order across participants, and our primary comparisons being between drug and placebo rather than across tasks.

Furthermore, buspirone possesses other pharmacological actions that may be involved in the results seen here. For example, buspirone has both partial agonist and antagonist effects at dopamine D_2_ receptors, which produce complex effects on dopamine neurotransmission. Thus, pre-clinical evidence indicates that acute buspirone administration increases dopamine release in the striatum (Algeri et al., 1988), with only mild post-synaptic dopamine antagonism (Dhavalshankh et al., 2007). This action could mitigate the impact of reduced reward sensitivity seen with a reduction in global serotonin activity brought about by buspirone (Seymour et al., 2012).

Healthy volunteer studies have used pramipexole, a dopamine D_2_/D_3_ receptor agonist, and sulpiride, a dopamine D_2_ receptor antagonist, to investigate the subjective rating and neural correlates of rewarding and aversive tastes (McCabe et al., 2011, 2012). These studies found no changes in subjective ratings of taste, which contrasts with the effect on bitterness seen here with buspirone.

## Conclusion

This study demonstrates the impact of acute buspirone on various stages of reward processing in healthy volunteers. Buspirone reduces the subjective experience of taste, particularly aversive taste, and maintains a sensitivity to effort when deciding whether to accept a reward in an effort-discounting task. Finally, buspirone increases sensitivity to aversive loss learning. Whilst buspirone possesses a complex pharmacology with affinity for dopamine receptors and partial agonism of post-synaptic 5-HT_1A_ receptors, its major acute pharmacological action is likely to be activation of somatodendritic 5-HT_1A_ receptors leading to reduction in serotonin activity at sites proximal to midbrain serotonergic neurones, such as the striatum and frontal cortex. Therefore, a speculative but plausible hypothesis is that a reduction in serotonin in these regions may increase aversion to primary stimuli and effort in aversive learning.

This effect could inform clinical discussion around possible increased sensitivity to negative events in the early stages of drug treatment, which involve 5-HT_1A_ agonism either directly, such as vortioxetine, or indirectly, such as SSRIs. However, this would be with the caveat that whilst 5-HT_1A_ agonism may be a common action to several serotonergic drugs, it would not be the only action, with other ‘non-5HT_1A_ receptor’ effects contributing to the early effects of such drug treatments.

## Supplemental Material

sj-docx-1-jop-10.1177_02698811261430527 – Supplemental material for The behavioural effects of the serotonin 1A receptor agonist buspirone on reward processing in healthy volunteers: A randomised, placebo-controlled study Supplemental material, sj-docx-1-jop-10.1177_02698811261430527 for The behavioural effects of the serotonin 1A receptor agonist buspirone on reward processing in healthy volunteers: A randomised, placebo-controlled study by Alexander L. W. Smith, Sorcha Hamilton, Susannah E. Murphy, Philip J. Cowen and Catherine J. Harmer in Journal of Psychopharmacology
