# Supplementary material for: The behavioural effects of the serotonin 1A receptor agonist buspirone on reward processing in healthy volunteers: A randomised, placebo-controlled study
Source: J Psychopharmacol. 2026 Mar 28;40(9):1381–91. doi: 10.1177/02698811261430527 (PMC13490652; doi:10.1177/02698811261430527)
Supplement: Supplementary file 1 [file sj-docx-1-jop-10.1177_02698811261430527.docx]

Smith ALW et al.

Supplementary material

**Supplementary text 1.**

Inclusion criteria

- *Participant is willing and able to give informed consent for participation in the research*
- *Male or female*

Exclusion criteria

*The participant may not enter the study if ANY of the following apply:*

- *Any current Axis 1 DSM-5 psychiatric disorder*
- *Any previous episode of a severe mental illness,*
- *A first degree relative diagnosed with Bipolar Affective Disorder Type 1 or Schizophrenia*
- *Body Mass Index less than 17 or deemed unacceptable by the medically-qualified team member on grounds of safety or scientific integrity*
- *Any significant current medical condition likely to interfere with conduct of the study or analysis of data (epilepsy, renal disease, hepatic disease, myasthenia gravis, acute closed-angle glaucoma)*
- *Current use of psychoactive and / or medically significant medication as judged by a medically-qualified team member, whether prescribed or bought over the counter (the contraceptive pill, the Depo-Provera injection or the progesterone implant* *will not result in exclusion)*
- *Past history of dependence to illicit substances, and any consumption of illicit substances in the three months prior to the study*
- *Currently pregnant or breast feeding*
- *Known lactase deficiency or any other problem absorbing lactose, galactose, or glucose*
- *Participation in a study using the same tasks in the last year*
- *Any physical (including visual and auditory) or language impairment that would make complying with the study protocol challenging. This includes any taste/olfactory disturbance e.g. secondary to Covid-19 infection*

Sample size calculation

Sample size calculation

Based on an effect size of response rate to buspirone in depression being 0.71 (Kishi et al. 2014). For a power of 0.9 to detect a significant difference between the two groups with an α of 5%. 28 participants in each group were required. To accommodate attrition, we aimed to recruit 31 participants in each group. Exclusion criteria included no contraindication to buspirone or no previous diagnoses of mental illness (full exclusion & inclusion criteria reported in supplementary material of (Smith et al. 2025)). Participants who, for whatever reason, were found to be ineligible for inclusion in the final dataset were removed and replaced with recruitment of additional participants.

A noted limitation of the effect size calculation presented here is that it is based upon responses of MDD subjects using clinical questionnaires, which may not have direct translatability to measuring responses to behavioural tasks in healthy volunteers. Results should be interpreted in this context.

**Supplementary text 2: Apples task logistic regression model rationale**

We analysed trial-by-trial “Yes” responses using mixed-effects logistic regression (using lme4::glmer in R) with stake and effort (z-standardised) and their interaction with allocation as fixed effects. This was chosen because each subject completed many trials and as we expected individual differences with regards to sensitivity to stake and effort, we compared several random-effects structures (see table below)

| Model | AIC | BIC | Log-Likelihood | Deviance | Likelihood ratio χ² | Df | p-value |
| --- | --- | --- | --- | --- | --- | --- | --- |
| Yes ~ stake_z * effort_z * allocation + (1 \| id) | 3651.3 | 3711.8 | -1816.7 | 3633.3 | NA | NA | NA |
| Yes ~ stake_z * effort_z * allocation +  (1 + stake_z \| id) | 3528.3 | 3602.2 | -1753.2 | 3506.3 | 127.0 | 2 | < 0.001 |
| Yes ~ stake_z * effort_z * allocation +  (1 + stake_z + effort_z \| id) | 3452.3 | 3546.4 | -1712.2 | 3424.3 | 82.0 | 3 | < 0.001 |
| Yes ~ stake_z * effort_z * allocation +  (1 + stake_z * effort_z \| id) | 3413.2 | 3534.1 | -1688.6 | 3377.2 | 47.2 | 4 | < 0.001 |

Likelihood-ratio tests and information criteria indicated that each increase in random-effects complexity significantly improved model. The final model therefore provided the best fit to the data (lowest AIC/highest log likelihood) while allowing subject-specific intercepts and slopes for stake, effort and their interaction.

We also checked whether the random-effects part of the model was too complex for the data (using performance::check_singularity in R) and found no evidence of this.

Supplementary table 1: CONSORT checklist

| **Section/ Topic** | **Item No** | **Checklist item** | **Reported on page No** |
| --- | --- | --- | --- |
| **Title and abstract** | | | |
|  | 1a | Identification as a randomised trial in the title | 1 |
|  | 1b | Structured summary of trial design, methods, results, and conclusions (for specific guidance see CONSORT for abstracts) | 1 |
| **Introduction** | | | |
| Background and objectives | 2a | Scientific background and explanation of rationale | 2-3 |
|  | 2b | Specific objectives or hypotheses | 3 |
| **Methods** | | | |
| Trial design | 3a | Description of trial design (such as parallel, factorial) including allocation ratio | 3-4 |
|  | 3b | Important changes to methods after trial commencement (such as eligibility criteria), with reasons | n/a |
| Participants | 4a | Eligibility criteria for participants | supplementary |
|  | 4b | Settings and locations where the data were collected | 4 |
| Interventions | 5 | The interventions for each group with sufficient details to allow replication, including how and when they were actually administered | 3 |
| Outcomes | 6a | Completely defined pre-specified primary and secondary outcome measures, including how and when they were assessed | 5 - 6 |
|  | 6b | Any changes to trial outcomes after the trial commenced, with reasons | n/a |
| Sample size | 7a | How sample size was determined | supplementary |
|  | 7b | When applicable, explanation of any interim analyses and stopping guidelines | n/a |
| Randomisation: |  |  |  |
| Sequence generation | 8a | Method used to generate the random allocation sequence | 4 |
|  | 8b | Type of randomisation; details of any restriction (such as blocking and block size) | 4 |
| Allocation concealment mechanism | 9 | Mechanism used to implement the random allocation sequence (such as sequentially numbered containers), describing any steps taken to conceal the sequence until interventions were assigned | 3 |
| Implementation | 10 | Who generated the random allocation sequence, who enrolled participants, and who assigned participants to interventions | 4 |
| Blinding | 11a | If done, who was blinded after assignment to interventions (for example, participants, care providers, those assessing outcomes) and how | 4 |
|  | 11b | If relevant, description of the similarity of interventions | n/a |
| Statistical methods | 12a | Statistical methods used to compare groups for primary and secondary outcomes | 6 |
|  | 12b | Methods for additional analyses, such as subgroup analyses and adjusted analyses | nil |
| **Results** | | | |
| Participant flow (a diagram is strongly recommended) | 13a | For each group, the numbers of participants who were randomly assigned, received intended treatment, and were analysed for the primary outcome | Figure 1 |
|  | 13b | For each group, losses and exclusions after randomisation, together with reasons | 7 & Figure 1 |
| Recruitment | 14a | Dates defining the periods of recruitment and follow-up | 4 |
|  | 14b | Why the trial ended or was stopped | 4 |
| Baseline data | 15 | A table showing baseline demographic and clinical characteristics for each group | 2 |
| Numbers analysed | 16 | For each group, number of participants (denominator) included in each analysis and whether the analysis was by original assigned groups | 7 |
| Outcomes and estimation | 17a | For each primary and secondary outcome, results for each group, and the estimated effect size and its precision (such as 95% confidence interval) | 8-12 |
|  | 17b | For binary outcomes, presentation of both absolute and relative effect sizes is recommended | 10 |
| Ancillary analyses | 18 | Results of any other analyses performed, including subgroup analyses and adjusted analyses, distinguishing pre-specified from exploratory | nil |
| Harms | 19 | All important harms or unintended effects in each group (for specific guidance see CONSORT for harms) | 7 |
| **Discussion** | | | |
| Limitations | 20 | Trial limitations, addressing sources of potential bias, imprecision, and, if relevant, multiplicity of analyses | 16 |
| Generalisability | 21 | Generalisability (external validity, applicability) of the trial findings | 13-17 |
| Interpretation | 22 | Interpretation consistent with results, balancing benefits and harms, and considering other relevant evidence | 13-17 |
| **Other information** | | |  |
| Registration | 23 | Registration number and name of trial registry | 1 |
| Protocol | 24 | Where the full trial protocol can be accessed, if available | 20 |
| Funding | 25 | Sources of funding and other support (such as supply of drugs), role of funders | 20 |

Citation: Schulz KF, Altman DG, Moher D, for the CONSORT Group. CONSORT 2010 Statement: updated guidelines for reporting parallel group randomised trials. BMC Medicine. 2010;8:18.
© 2010 Schulz et al. This is an Open Access article distributed under the terms of the Creative Commons Attribution License (<http://creativecommons.org/licenses/by/2.0>), which permits unrestricted use, distribution, and reproduction in any medium, provided the original work is properly cited.

| Supplementary Table 2: Demographics and psychometrics of participants | | |
| --- | --- | --- |
|  | Buspirone (N = 31) | Placebo (N = 31) |
| Gender, N female:male | 17:14 | 17:14 |
| Age (years), mean (S.D.) | 25.19 (6.00) | 27.28 (8.72) |
| BDI^a^, mean (S.D.) | 3.03 (3.03) | 2.19 (2.71) |
| ^a^ BDI = Becks Depression Inventory | | |

**Supplementary table 3**

Table detailing different model of regression with AIC, BIC and R^2^ values to identify the most parsimonious model with the best fitting model for the data. Models were created for separate tastes on the hypothesis of each taste using distinct neural pathways e.g. sour and bitter tastes utilising aversive neural pathways, whereas sweet tastes utilising reward neural pathways.

| **Taste** | **Formula** | **Adj. R^2^** | **R SE** | **AIC** | **BIC** |
| --- | --- | --- | --- | --- | --- |
| Sweet | post_Pleasure ~ pre_Pleasure + Nausea2 + allocation * post_Intensity * post_Anticipation | 0.6222 | 11.29 | 410.5945 | 432.0582 |
| Sweet1 | post_Pleasure ~ pre_Pleasure + allocation + Nausea2 + post_Intensity * post_Anticipation | 0.5679 | 12.08 | 415.1665 | 430.7765 |
| Sweet2 | post_Pleasure ~ pre_Pleasure + allocation + Nausea2 + post_Intensity + post_Anticipation | 0.5596 | 12.20 | 415.3057 | 428.9644 |
| Sweet3 | post_Pleasure ~ pre_Pleasure + allocation * post_Intensity * post_Anticipation | 0.5934 | 11.72 | 413.6397 | 433.1521 |
| Sweet4 | post_Pleasure ~ pre_Pleasure + allocation + post_Intensity * post_Anticipation | 0.4923 | 13.09 | 422.6964 | 436.3551 |
| Sweet5 | post_Pleasure ~ pre_Pleasure + allocation + post_Intensity + post_Anticipation | 0.4734 | 13.34 | 423.7212 | 435.4286 |
| Sour | post_Pleasure ~ pre_Pleasure + Nausea2 + allocation * post_Intensity * post_Anticipation | 0.762 | 10.61 | 404.134 | 425.598 |
| Sour1 | post_Pleasure ~ pre_Pleasure + allocation + Nausea2 + post_Intensity * post_Anticipation | 0.736 | 11.18 | 407.147 | 422.756 |
| Sour2 | post_Pleasure ~ pre_Pleasure + allocation + Nausea2 + post_Intensity + post_Anticipation | 0.741 | 11.06 | 405.186 | 418.845 |
| Sour3 | post_Pleasure ~ pre_Pleasure + allocation * post_Intensity * post_Anticipation | 0.753 | 10.81 | 405.210 | 424.722 |
| Sour4 | post_Pleasure ~ pre_Pleasure + allocation + post_Intensity * post_Anticipation | 0.704 | 11.82 | 412.084 | 425.743 |
| Sour5 | post_Pleasure ~ pre_Pleasure + allocation + post_Intensity + post_Anticipation | 0.710 | 11.72 | 410.256 | 421.963 |
| Salt | post_Pleasure ~ pre_Pleasure + Nausea2 + allocation * post_Intensity * post_Anticipation | 0.5402 | 12.57 | 421.7451 | 443.2088 |
| Salt1 | post_Pleasure ~ pre_Pleasure + allocation + Nausea2 + post_Intensity * post_Anticipation) | 0.5584 | 12.32 | 417.2300 | 432.8400 |
| Salt2 | post_Pleasure ~ pre_Pleasure + allocation + Nausea2 + post_Intensity + post_Anticipation) | 0.561 | 12.29 | 416.0689 | 429.7277 |
| Salt3 | post_Pleasure ~ pre_Pleasure + allocation * post_Intensity * post_Anticipation) | 0.5509 | 12.43 | 419.7453 | 439.2578 |
| Salt4 | post_Pleasure ~ pre_Pleasure + allocation + post_Intensity * post_Anticipation) | 0.5674 | 12.2 | 415.3053 | 428.9640 |
| Salt5 | post_Pleasure ~ pre_Pleasure + allocation + post_Intensity + post_Anticipation) | 0.5701 | 12.16 | 414.1001 | 425.8076 |
| Bitter | bitter_model ~ pre_Pleasure + Nausea2 + allocation * post_Intensity * post_Anticipation | 0.533 | 11.77 | 414.85 | 436.32 |
| Bitter1 | post_Pleasure ~ pre_Pleasure + allocation + Nausea2 + post_Intensity * post_Anticipation | 0.523 | 11.89 | 413.50 | 429.11 |
| Bitter2 | post_Pleasure ~ pre_Pleasure + allocation + Nausea2 + post_Intensity + post_Anticipation | 0.514 | 12.01 | 413.67 | 427.33 |
| Bitter3 | post_Pleasure ~ pre_Pleasure + allocation * post_Intensity * post_Anticipation | 0.536 | 11.73 | 413.77 | 433.28 |
| Bitter4 | post_Pleasure ~ pre_Pleasure + allocation + post_Intensity * post_Anticipation | 0.532 | 11.78 | 411.69 | 425.35 |
| Bitter5 | post_Pleasure ~ pre_Pleasure + allocation + post_Intensity + post_Anticipation | 0.522 | 11.90 | 411.91 | 423.62 |

Supplementary tables 4

| Anticipation | | | |
| --- | --- | --- | --- |
| Taste | run | Buspirone mean (st. dev) | Placebo mean (st. dev) |
| Bitter | pre | -11.41 (21.48) | -16.81 (22.70) |
|  | post | -11.41 (20.15) | -18.07 (20.87) |
| Salt | pre | 12.37 (20.55) | 14.48 (23.43) |
|  | post | 11.04 (17.19) | 14.37 (19.84) |
| Sour | pre | 11.81 (20.47) | -1.00 (19.37) |
|  | post | 16.44 (14.29) | 3.52 (23.89) |
| Sweet | pre | 29.93 (16.47) | 30.48 (16.13) |
|  | post | 23.07 (17.01) | 27.78 (17.33) |

| Intensity | | | |
| --- | --- | --- | --- |
| Taste | run | Buspirone mean (st. dev) | Placebo mean (st. dev) |
| Bitter | pre | -14.22 (25.26) | -19.19 (20.69) |
|  | post | -11.70 (27.91) | -11.15 (22.30) |
| Salt | pre | -10.56 (17.21) | -12.19 (19.09) |
|  | post | -6.59 (19.66) | -11.37 (21.53) |
| Sour | pre | -3.22 (17.32) | -5.44 (19.06) |
|  | post | 3.07 (21.24) | 0.67 (20.99) |
| Sweet | pre | -24.37 (12.29) | -23.00 (13.14) |
|  | post | -17.48 (21.36) | -15.81 (17.69) |

| Pleasure | | | |
| --- | --- | --- | --- |
| Taste | run | Buspirone mean (st dev.) | Placebo mean (st. dev) |
| Bitter | pre | -13.89 (16.48) | -16.89 (17.31) |
|  | post | -19.04 (17.35) | -15.04 (18.08) |
| Salt | pre | 9.48 (13.84) | 5.19 (19.13) |
|  | post | 10.00 (15.58) | 8.04 (20.82) |
| Sour | pre | 16.93 (16.46) | 5.44 (20.12) |
|  | post | 16.59 (15.86) | 5.74 (24.90) |
| Sweet | pre | 15.37 (13.65) | 18.41 (12.71) |
|  | post | 14.67 (21.41) | 26.44 (11.54) |

Kishi, T., H. Y. Meltzer, Y. Matsuda, and N. Iwata. 2014. 'Azapirone 5-HT1A receptor partial agonist treatment for major depressive disorder: systematic review and meta-analysis', *Psychol Med*, 44: 2255-69.

Smith, Alexander L. W., Sorcha Hamilton, Susannah E. Murphy, Philip J. Cowen, and Catherine J. Harmer. 2025. 'The behavioural effects of the serotonin 1A receptor agonist buspirone on cognition and emotional processing in healthy volunteers', *Psychopharmacology*.
